# Separation Time of Aluminothermic Reduction Products for Sustainable Silicon Production

**DOI:** 10.12688/openreseurope.18833.1

**Published:** 2024-11-20

**Authors:** Javier Bullón, Óscar Crego, José Luis Ferrín, Dolores Gómez, Iván Martínez, Luis Javier Pérez-Pérez

**Affiliations:** 1 SilBuCam, A Coruña, Spain; 2 Centro de Investigación e Tecnoloxía Matemática de Galicia, Santiago de Compostela, Galicia, 15782, Spain; 3 Universidade de Santiago de Compostela Departamento de Matematica Aplicada, Santiago de Compostela, Galicia, 15782, Spain; 4 Universidad Politecnica de Madrid Departamento de Ingenieria Geologica y Minera, Madrid, Community of Madrid, 28003, Spain

**Keywords:** mathematical modelling, numerical simulation, rotary kiln, aluminothermic reduction, multiphase flow, separation time

## Abstract

**Background:**

This work was carried out within the framework of the SisAl Pilot project, which is devoted to the environmentally friendly production of silicon. This new method relies on the aluminothermic reduction of quartz in slag, offering a more sustainable alternative to the traditional reduction of silica with carbon in submerged arc furnaces.

**Methods:**

The process takes place in a rotary kiln producing silicon (Si) and alumina slag (actually, a CaO – Al _2_O _3_ slag), which must be separated at the end to extract the silicon. This separation process is analyzed through mathematical modelling and numerical simulation, as it is of industrial interest to know how much time it takes for Si and CaO – Al _2_O _3_ slag to separate once the process has ended. Generally, a multiphase flow model is used to estimate the separation time of the two components once aluminothermic reduction has ended.

**Results:**

Several scenarios are considered for the numerical simulation of the separation time, namely different initial configurations and material properties of both fluids are covered. Moreover, the separation times obtained with two distinct multiphase flow models -VOF (volume of fluid) and Eulerian- are compared.

**Conclusions:**

The separation times resulting from simulations using the multiphase Eulerian model are more realistic compared to those from the VOF model, which clearly tends to underestimate separation times. Furthermore, apart from the selected multiphase flow model, the density difference between silicon and alumina slag plays a critical role in determining the separation time.

## Plain language summary

The traditional production of silicon involves a large amount of carbon dioxide emissions into the atmosphere. To improve this situation, the SisAl Pilot project proposes a new, eco-friendly method that aims to produce silicon and alumina slag as a by-product in a more sustainable manner. This work utilizes mathematical modelling and numerical simulation to estimate the separation time of silicon from slag, with the goal of extracting only high-purity silicon.

## 1. Introduction

Silicon is a crucial material in European industries, and its production is of great significance to many countries. Traditionally, silicon has been produced by reducing silica with carbon in submerged arc furnaces (SAF) ^1^. However, the aluminothermic reduction process proposed by the SisAl Pilot project ^2, 3^ presents a more environmentally friendly alternative, as it eliminates the need for raw carbon materials, thereby lowering CO _2_ emissions. The SisAl Pilot project is exploring this method to produce decarbonized silicon while also recycling aluminum from dross and scrap. This process can be carried out in different types of furnaces, including electric arc, induction, and rotary kilns. The last type is considered in this study, namely, a gas rotary furnace placed at Fundiciones Rey (FRey) facilities in Spain.

In the aluminothermic reduction process, the SisAl reaction mechanism takes place:

$${\text{SiO}}_{2}(\text{CaO}-{\text{SiO}}_{2}\;\text{slag})+\frac{4}{3}\text{Al}\to \text{Si}+\frac{2}{3}{\text{Al}}_{2}{\text{O}}_{3}(\text{CaO}-{\text{Al}}_{2}{\text{O}}_{3}\;\text{slag}).$$

As a result of the aluminothermic reduction, Si and CaO – Al _2_O _3_ slag are obtained, and they need to be separated to obtain the purest possible silicon. The main goal of this work was to estimate the time required for both fluids to completely separate after the end of the aluminothermic reduction. To achieve this objective, the separation process was mathematically modelled according to the Navier-Stokes equations and adequate multiphase flow models. Then, numerical simulations of the separation process were carried out under various scenarios, including different initial conditions, material properties, and multiphase models.

This paper is organized as follows. In Section 2, the methodology followed through this work is presented, including a description of the industrial problem, the geometry of the rotary furnace and the physical properties of the materials involved. The proposed mathematical model is also described. Section 3 presents the main numerical results. Finally, in Section 4, conclusions regarding the obtained results are presented.

## 2. Methods

In this section, the methodology followed in this work is detailed. More precisely, the industrial problem related to the aluminothermic reduction in the rotary kiln and the mathematical modelling proposed to estimate the separation time are described.

### Problem description

**Figure 1.**
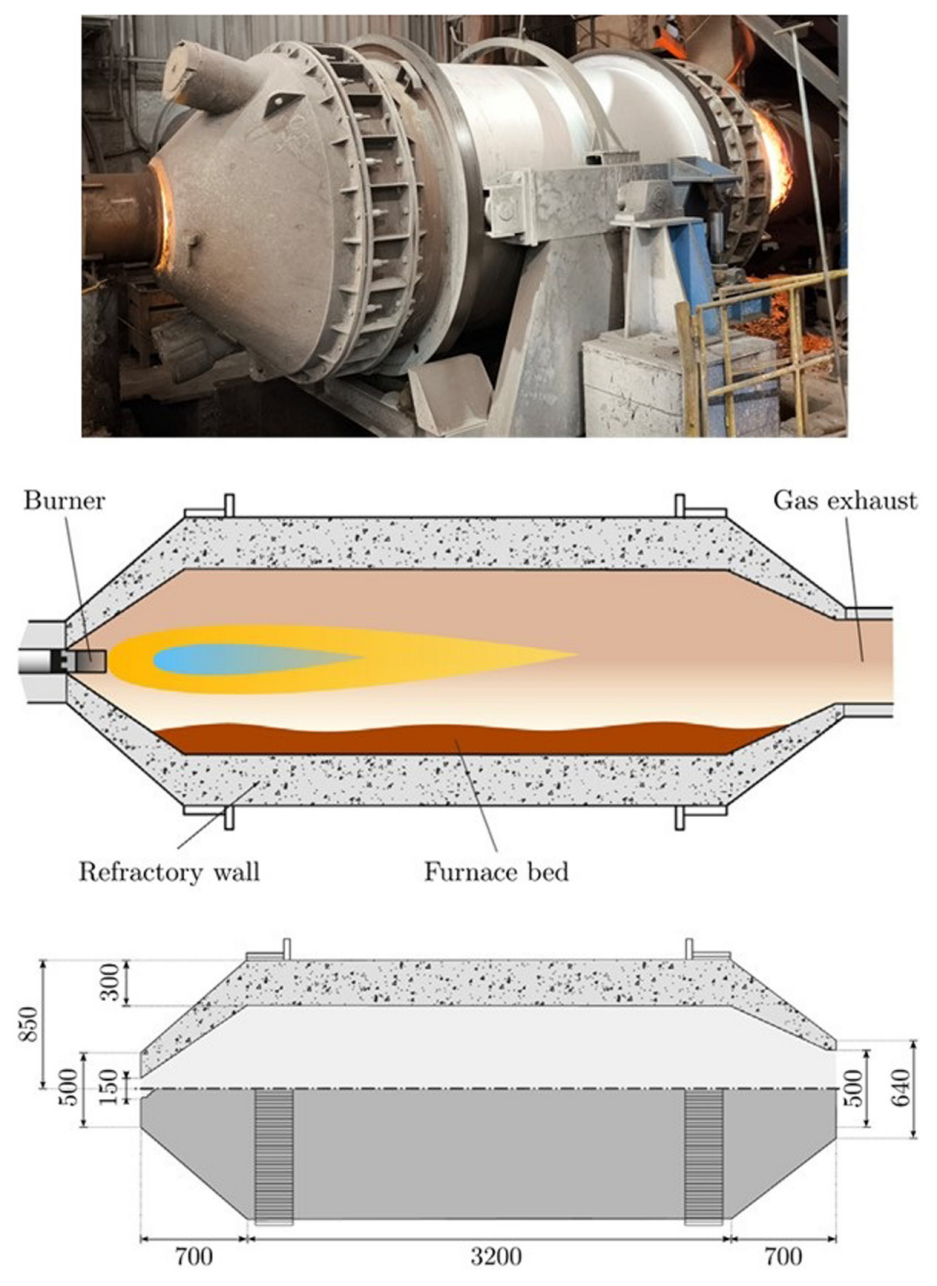
Rotary furnace at FRey (top), schematic diagram (center) and dimensions in mm (bottom).

The gas rotary furnace considered in this study is made of a cylindrical steel vessel lined with a thick refractory coating, which rolls around its axial direction following two turned rings that act as guides. To enhance the mixing and homogenize the temperature of the partially molten materials, the furnace can be rotated using an electrical motor. A longitudinal view of the furnace and its main dimensions are shown in Figure 1.

During operation, on one side of the furnace, the exhaust gas collection hood remained open, and the opposite side was closed with a lid that included an oxy-fuel burner in its center. The burner injected natural gas and oxygen to generate an intense flame. The furnace can be tilted to facilitate the loading of the solid material and discharge of the melted material once the melting process is completed.

The simulation of the separation time assumes that the aluminothermic reduction has finished; therefore, neither combustion inside the furnace nor slag melting were considered in this process. Once the reaction had ended, a multiphase flow with two fluids, Si and CaO – Al _2_O _3_, was considered, and their evolution over time was analyzed.

**Figure 2.**
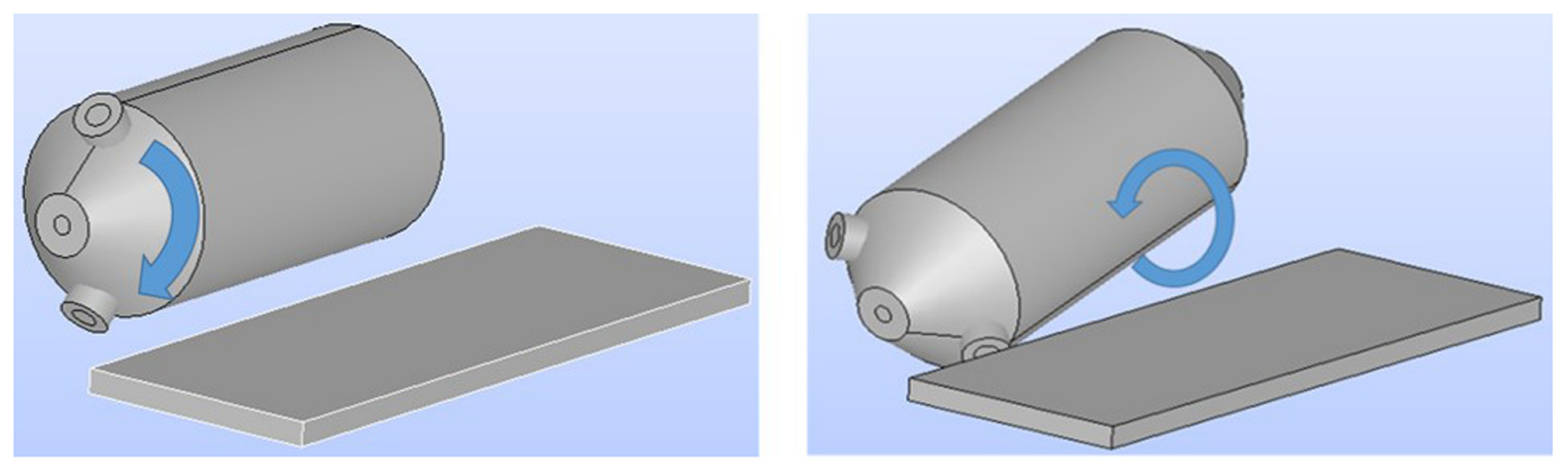
Possible rotation movements of the rotary furnace.

First, the geometry of the load in the rotary furnace was determined. This is the computational domain of the simulation, which mainly depends on the inclination angle of the furnace and burden volume. The two possible rotation movements of the furnace during operation are depicted in Figure 2.

**Figure 3.**
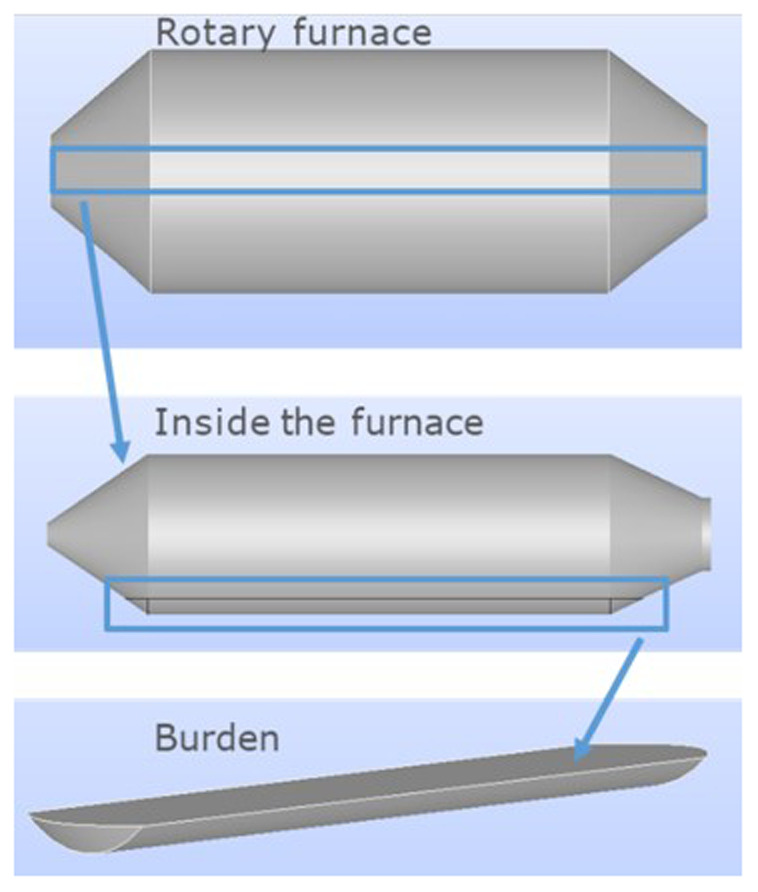
Location of the computational domain inside the rotary furnace.

To determine the position of the charge, the relevant rotation angle was measured with respect to the horizontal plane, as the rotation along the largest furnace dimension was not considered. Thus, once the rotation with respect to the horizontal plane and burden volume are given, the geometry of the charge can be obtained. Note that the computational domain is the charge inside the furnace, not including air or the furnace itself, as shown in Figure 3.

**Figure 4.**
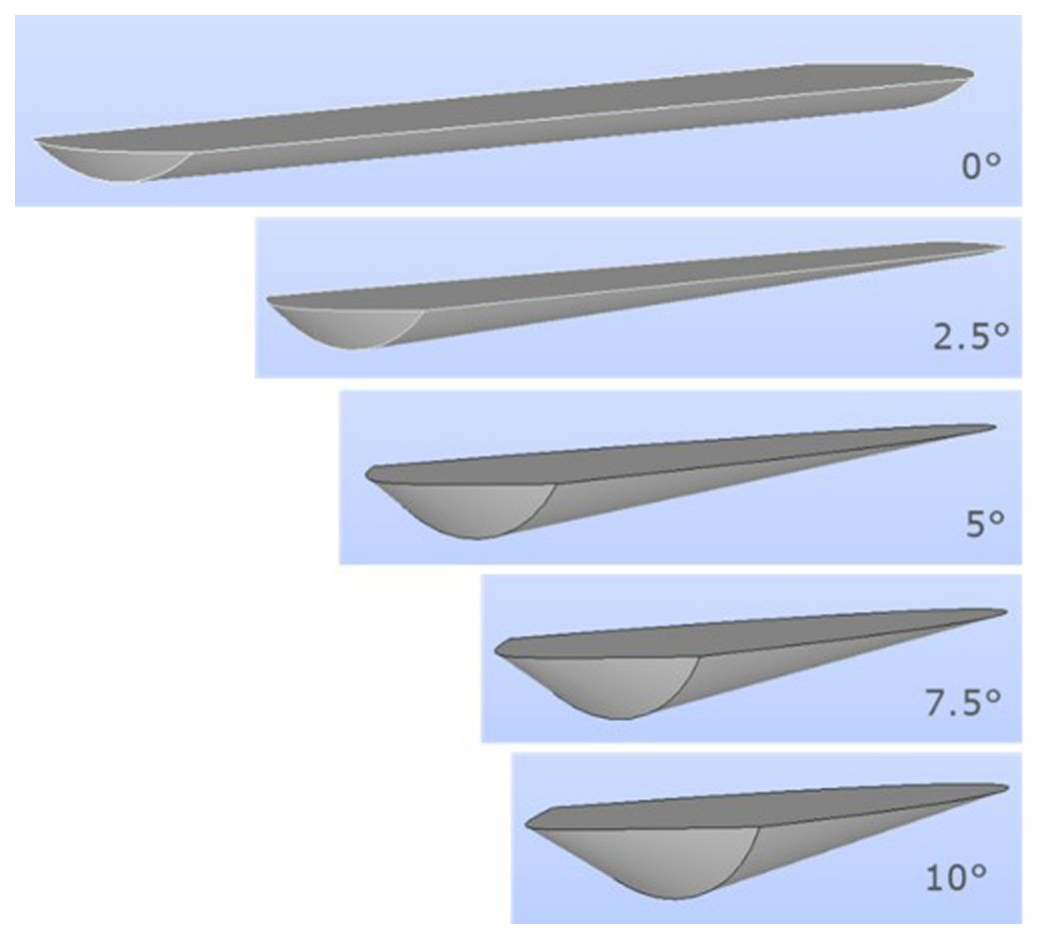
Charge geometry depending on the rotation angle for a given burden volume.

Several charge geometries for different rotation angles are shown in Figure 4. The charge geometry used in the simulations corresponded to an inclination of 5°.

**Figure 5.**
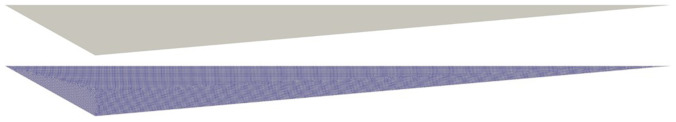
Computational domain (top) and mesh (bottom).

In principle, a 3D simulation considering Si, CaO – Al _2_O _3_ and air is desirable; however, the computational cost makes it unaffordable. The problem can be modelled, in a very good approximation, by a 2D model and by only considering the geometry of the charge corresponding to Si and CaO – Al _2_O _3_ in a longitudinal cut of the 3D domain. The proposed 2D computational domain and corresponding finite volume mesh for an inclination of 5° are shown in Figure 5.

The physical properties needed for Si and CaO – Al _2_O _3_ are density and viscosity, which are summarized in Table 1.

**Table 1.** Densities and viscosities considered for Si and CaO – Al _2_O _3_ in the simulations.

	Si	CaO – Al _2_O _3_
Density, ***ρ*** (kg m ^-3^)	3240	2500
Viscosity, ***μ*** (Pa s)	5.82E-4	0.1353

Note that both the density and viscosity of the silicon were modified to account for the fact that the furnace is commonly used to melt iron at FRey facilities; therefore, the final slag composition would be contaminated by iron adhering to the furnace walls.

### Mathematical modelling

The separation problem within the charge domain was modelled using a transient hydrodynamic model. Specifically, the averaged Navier-Stokes equations and the K – ω SST turbulence model are considered in addition to an adequate multiphase flow model. The mass and momentum conservation equations are given by:

div(v)=0,

$$\frac{\partial }{\partial t}(\rho \text{v})+\text{div}(\rho \text{v}⊗\text{v})=-\text{grad}(p)-\text{div}\left((\mu +\mu_{T})\left[2\text{D}(\text{v})-\frac{2}{3}\text{div}(\text{v})\text{I}\right]-\frac{2}{3}\rho K\text{I}\right),$$

where **v** is the fluid velocity, *p* is the pressure, *μ _T_* is the turbulent viscosity, **D**( **v**) is the symmetric part of the velocity gradient, and **I** is the identity tensor.

Moreover, the K – ω SST turbulence model ^4, 5^ involves two additonal equations for the turbulent kinetic energy, *K*, and the specific rate of dissipation, *ω*:

$$\frac{\partial }{\partial t}(\rho K)+\text{div}(\rho K\text{v})=\text{div}({Г}_{K}\text{grad}K)+{\tilde{G}}_{K}-Y_{K},$$

$$\frac{\partial }{\partial t}(\rho \omega )+\text{div}(\rho \omega \text{v})=\text{div}({Г}_{\omega }\text{grad}\omega )+G_{\omega }-Y_{\omega }+D_{\omega }.$$

In the previous equations, ${\tilde{G}}_{K}$ represents the generation of turbulence kinetic energy due to mean velocity gradients, *G _ω_* represents the generation of *ω*; *Y _K_* and *Y _ω_* represent the dissipation of *K* and *ω*; Γ *_K_* and Γ *_ω_* represent the effective diffusivity of *K* and *ω*; and *D _ω_* is the cross-diffusion term.

With respect to the boundary conditions, zero-shear stress was considered on the top free surface, and no-slip conditions were considered on the walls.

In addition to the conservation equations and turbulence modelling, an adequate multiphase model is required to properly model the separation of both fluids. There are two main multiphase models that use different mathematical descriptions: the VOF model (see 6) and the Eulerian model. The VOF model can handle several immiscible fluids by solving a single set of momentum equations and by tracking the volume fraction of each fluid throughout the domain. On the other hand, the Eulerian option can also model several fluids but the momentum and continuity equations are considered for each phase separately. The VOF model is suitable for flows with a clearly defined phase interface, as both phases are non-interpenetrating and cannot be mixed. In contrast, the Eulerian model assumes that the two continuous phases form an interpenetrating continuum.

As a first approach, the VOF model is considered because its complexity and computational cost are less expensive than those of the Eulerian model. The general idea is to consider the initial distribution of Si and Al _2_O _3_ on the charge, simulate the evolution of both fluids until they are conveniently separated, and determine the time required to reach the final state. After this time, a denser fluid should be placed at the bottom of the charge.

## 3. Numerical results

The proposed model was solved using commercial software ANSYS Fluent 2022 R2 ^7^. It employs a cell-centered finite volume method to discretize the conservation equations. The coupling of the velocity and pressure was achieved using the SIMPLE iterative algorithm. Least-squares cell-based schemes are considered to handle the diffusive terms, whereas second-order upwind schemes are utilized for the treatment of convective terms. Pressure interpolation was performed using PRESTO! scheme. Time discretization was performed using a second-order implicit scheme. For a more in-depth understanding of these numerical schemes, interested readers are encouraged to refer to 8 for further details.

**Figure 6.**
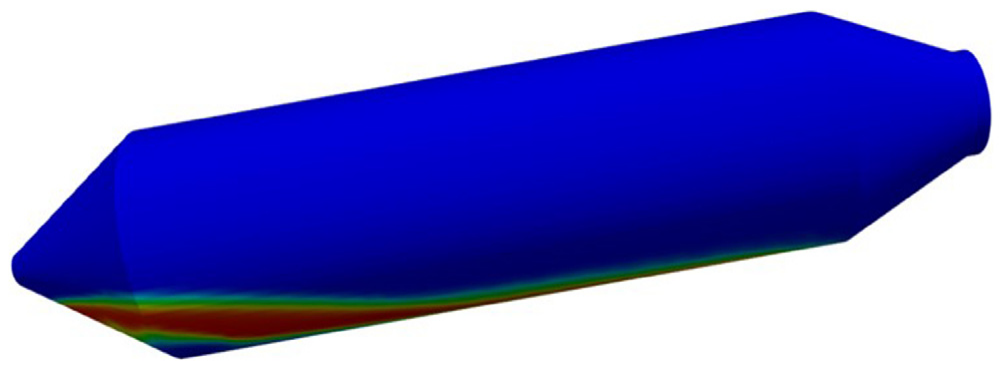
3D simulation, including air, Si and CaO – Al _2_O _3_ slag, which is computationally unaffordable.

Before the 2D resolution of the hydrodynamic model, a 3D test was considered only for demonstration purposes (see Figure 6). As expected, several weeks of computational time were required to advance only a few seconds of real-time simulation, despite the poor meshing considered.

Different scenarios were considered for the 2D simulations with respect to the multiphase flow model, material properties, and initial configurations of both fluids in the computational domain. The cases addressed in this study are summarized in Table 2.

**Table 2.** Characteristics of the different scenarios considered.

Scenario	Multiphase model	Initial configuration	Material properties
Case 1	VOF	Horizontal separation (Si on top)	Table 1
Case 2	VOF	Vertical separation	Table 1
Case 3	VOF	Horizontal separation (Si on top)	*μ* = 3.45 Pa s (CaO-Al _2_O _3_)
Case 4	VOF	Horizontal separation (Si on top)	*μ* = 3.45 × 10 ^–4^ Pa s (CaO-Al _2_O _3_)
Case 5	Eulerian	Horizontal separation (Si on top)	Table 1
Case 6	Eulerian	Homogeneous mixture	Table 1
Case 7	Eulerian	Homogeneous mixture	*ρ* = 2570 kg m ^-3^ (Si); *ρ* = 2600 kg m ^-3^ (CaO-Al _2_O _3_)

**Figure 7.**
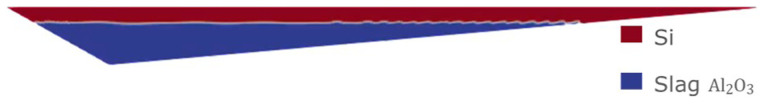
Case 1: initial configuration with Si on top.

First, the least favorable initial condition for the VOF model is considered, with a denser fluid (Si) on top at the initial time of the simulation, as shown in Figure 7.

**Figure 8.**
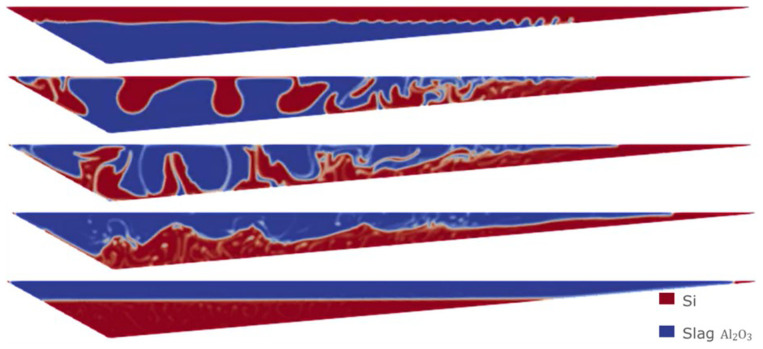
Case 1: complete separation after 30 s (bottom image).

The evolution of the simulation with the previous initial condition is shown in Figure 8, giving rise to a complete separation after 30 s.

**Figure 9.**
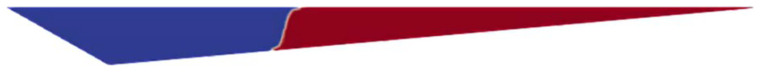
Case 2: initial configuration with vertical separation.

Different initial conditions can be considered, for instance, a vertical initial separation between the two fluids, as depicted in Figure 9.

**Figure 10.**
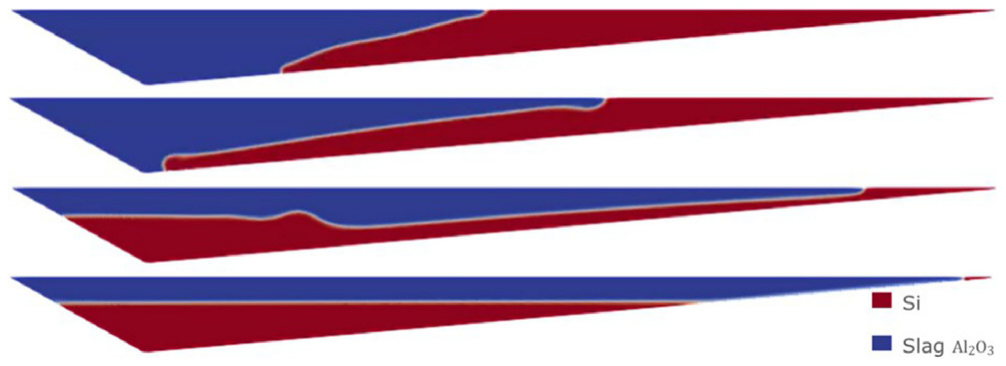
Case 2: complete separation after 30 s (bottom).

Although the initial condition is different from that of Case 1, the evolution of the simulation ends with a complete separation after 30 s, as shown in Figure 10.

**Figure 11.**
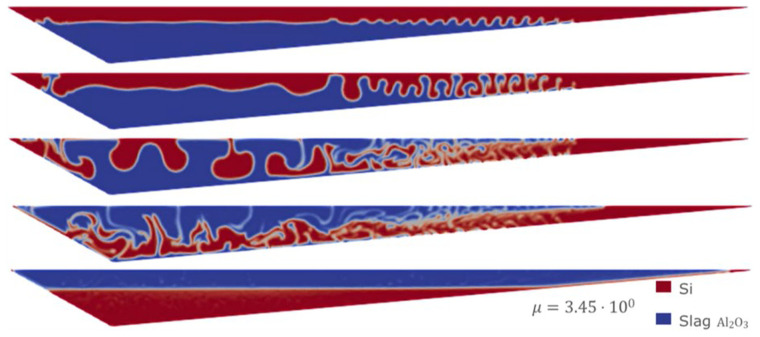
Case 3: complete separation after 30 s (bottom) for *μ _slag_* = 3.45 Pa s.

**Figure 12.**
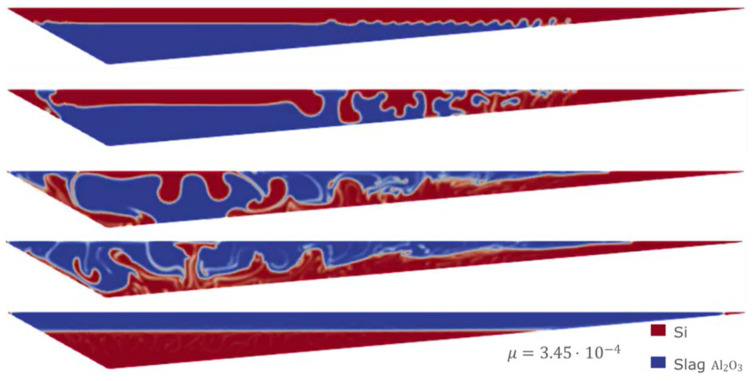
Case 4: complete separation after 30 s (bottom) for *μ _slag_* = 3.45×10 ^-4^ Pa s.

Sensitivity analyses were performed using the VOF model. In particular, starting from the least favorable initial condition (the same as in Case 1), extreme values for slag CaO – Al _2_O _3_ viscosity were considered, as this value is difficult to obtain from experimental measurements. In Figure 11 and Figure 12 the evolution for both Case 3 and Case 4 is shown, where different values of the slag CaO – Al _2_O _3_ viscosity were considered.

It is observed that in all the simulations carried out with the VOF model, both Si and CaO – Al _2_O _3_ are fully separated after 30 s, regardless of the initial conditions or viscosities. Because this separation time is much shorter than the expected time for this type of process, we will change the multiphase VOF model for the Eulerian model.

**Figure 13.**
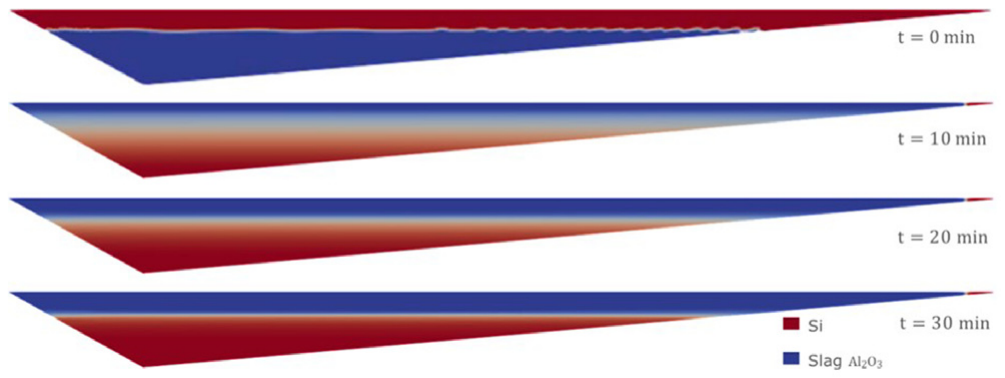
Case 5: complete separation after 30 min (bottom).

Therefore, we consider the horizontal initial condition of Case 1, but for the Eulerian model, where the denser fluid (Si) is on top at the initial time of the simulation. The evolution of the separation of the two fluids is shown in Figure 13. After 30 min of simulation, the two fluids were almost completely separated.

**Figure 14.**
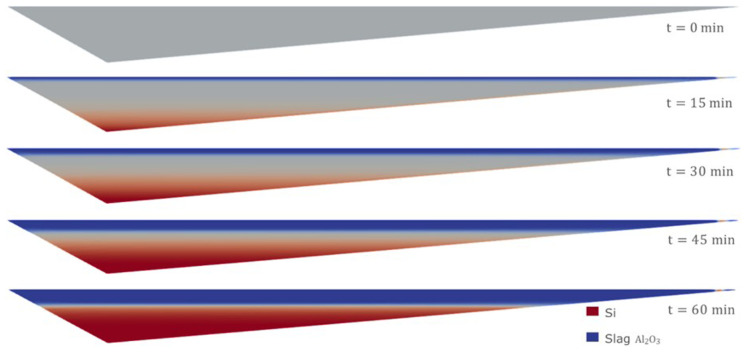
Case 6: complete separation after 60 min (bottom).

Moreover, using the Eulerian model, it is possible to consider an initial homogeneous mixture of Si and CaO – Al _2_O _3_. This is probably the most realistic and unfavorable initial condition for the separation process, and it cannot be considered when employing the VOF model. The evolution of the separation under this initial condition is shown in Figure 14. It was necessary to wait for at least 60 min to obtain an almost complete separation between Si and CaO – Al _2_O _3_.

The separation times resulting from the Eulerian model are much higher than those obtained with the VOF model, and seem more realistic according to the knowledge obtained from the plant experience.

**Figure 15.**
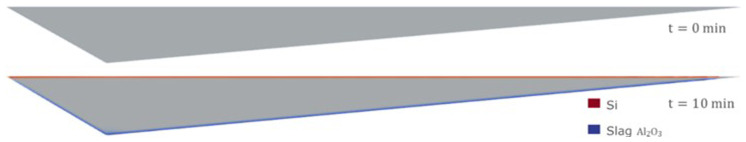
Case 7: no separation after 10 minutes when densities are almost equal.

However, it is well known that the difference in densities between Si and CaO – Al _2_O _3_ is the key factor in controlling the separation time, which is more important than any other parameter. This can be appreciated in the following simulation using the Eulerian model, starting from the homogeneous mixture condition and with the densities of both fluids modified to 2570 kg m ^-3^ for Si and 2600 kg m ^-3^ for CaO – Al _2_O _3_. As shown in Figure 15, after 10 min of simulation, almost no separation was observed. This is because of the similarity of both densities, which leads to extremely long separation times.

## 4. Conclusions

Once the aluminothermic reduction process in the rotary furnace has finished, it is of industrial interest to determine how long it will take for silicon and alumina slag to separate and ensure that only silicon is cast. To meet this requirement, own code was developed to calculate the geometry of the charge inside the furnace based on its geometry, inclination, and mass of charge. After determining the geometry, a multiphase flow model was applied to estimate the separation time of the two components after the reduction process. The separation time was simulated under different conditions including different material properties, initial conditions, and multiphase models. The results obtained with the multiphase Eulerian model are more realistic than those obtained with the VOF model, which has an obvious tendency to underestimate the separation times.

As is clear from the simulations, apart from the selected multiphase flow model, the density difference between silicon and alumina slag plays a critical role in determining the separation time.

## Ethics and consent

Ethics and consent were not required for this study.

## Data Availability

No data associated with this article. The numerical simulations in this work were performed using ANSYS Fluent 2022 R2. An open source alternative could be OpenFOAM ^9^, which is capable of doing similar tasks as those performed in this work.
