# Supplementary material for: Separation Time of Aluminothermic Reduction Products for Sustainable Silicon Production
Source: Open Res Eur. 2025 Jan 3;4:249. Originally published 2024 Nov 20. [Version 2] doi: 10.12688/openreseurope.18833.2 (PMC11724202; doi:10.12688/openreseurope.18833.2)
Supplement: Peer review history

# Reviewer report: Reviewer response for version 2

**DOI:** 10.21956/openreseurope.20838.r49487

**Published:** 2025-02-03

**Authors:** Mita Dasog

**Affiliations:** 1 Dalhousie University, Halifax, Nova Scotia, Canada

The study investigates the separation process of Si and CaO-Al _2_O _3_ slag in the SisAl Pilot project’s aluminothermic reduction method using mathematical modeling and numerical simulation. By comparing two multiphase flow models, the results indicate that the Eulerian model provides more realistic separation times than the VOF model, which tends to underestimate them. As alternatives to carbothermal reduction are currently being explored, it is important to demonstrate the commercial feasibility of alternative reduction methods.

 The paper can benefit from further clarifications and responses to the following queries:

 1. The temperature of the mixture was considered to be 1400 ^o^C, as obtained from the experimental work. Is this temperature measured at the surface of the mixture or assumed to be uniform throughout? Rotary kilns are known to have a radial thermal gradient, which is likely to be more pronounced during the separation step when the kiln is not rotating. If this temperature corresponds to the surface, how would a thermal gradient affect the viscosity and, consequently, the calculations?

 2.Can the authors explain why a 5-degree inclination was chosen for the simulations?  

 3.While the effect of viscosity was investigated using the VOF model, it was not examined with the Eulerian model. Can the authors clarify why this was the case?  

 4. A discussion on the accuracy or error margin in the time values predicted using the Eulerian model would be beneficial to readers.  

 5. There is a typographical error in Table 1: In the third column label, the numbers in Al _2_O _3_ should be subscripted.

Is the study design appropriate and does the work have academic merit?

Yes

Is the work clearly and accurately presented and does it cite the current literature?

Yes

If applicable, is the statistical analysis and its interpretation appropriate?

I cannot comment. A qualified statistician is required.

Are all the source data underlying the results available to ensure full reproducibility?

No source data required

Are the conclusions drawn adequately supported by the results?

Partly

Are sufficient details of methods and analysis provided to allow replication by others?

Yes

Reviewer Expertise:

NA

I confirm that I have read this submission and believe that I have an appropriate level of expertise to confirm that it is of an acceptable scientific standard, however I have significant reservations, as outlined above.

# Reviewer report: Reviewer response for version 2

**DOI:** 10.21956/openreseurope.20838.r49461

**Published:** 2025-01-10

**Authors:** Zulfiadi Zulhan

**Affiliations:** 1 Bandung Institute of Technology, Bandung, Indonesia

I have reviewed the manuscript, and I confirm that most of the revisions suggested have been implemented by the authors. Therefore, I recommend that the manuscript be accepted for indexing.

Is the study design appropriate and does the work have academic merit?

Partly

Is the work clearly and accurately presented and does it cite the current literature?

Partly

If applicable, is the statistical analysis and its interpretation appropriate?

Not applicable

Are all the source data underlying the results available to ensure full reproducibility?

Partly

Are the conclusions drawn adequately supported by the results?

Partly

Are sufficient details of methods and analysis provided to allow replication by others?

Partly

Reviewer Expertise:

NA

I confirm that I have read this submission and believe that I have an appropriate level of expertise to confirm that it is of an acceptable scientific standard.

# Reviewer report: Reviewer response for version 2

**DOI:** 10.21956/openreseurope.20838.r49462

**Published:** 2025-01-08

**Authors:** Navneet Singh Randhawa

**Affiliations:** 1 CSIR-National Metallurgical Laboratory, Jharkhand, India

The revised article may be accepted.

Is the study design appropriate and does the work have academic merit?

Yes

Is the work clearly and accurately presented and does it cite the current literature?

Partly

If applicable, is the statistical analysis and its interpretation appropriate?

I cannot comment. A qualified statistician is required.

Are all the source data underlying the results available to ensure full reproducibility?

No

Are the conclusions drawn adequately supported by the results?

Yes

Are sufficient details of methods and analysis provided to allow replication by others?

Partly

Reviewer Expertise:

NA

I confirm that I have read this submission and believe that I have an appropriate level of expertise to confirm that it is of an acceptable scientific standard.

# Reviewer report: Reviewer response for version 1

**DOI:** 10.21956/openreseurope.20381.r46985

**Published:** 2024-12-18

**Authors:** Zulfiadi Zulhan

**Affiliations:** 1 Bandung Institute of Technology, Bandung, Indonesia

The manuscript is part of the SisAl project to produce environmentally friendly silicon metal using SiO₂-CaO slag as raw material. The manuscript focuses on the separation of silicon from CaO-Al₂O₃ slag.

 However, the manuscript does not provide information regarding the temperature of the silicon separation process and the CaO-Al₂O₃ slag, including viscosity and density data of silicon and CaO-Al₂O₃ slag at what temperature? What are the data sources for Table 1 and Table 2?

 What was the environmental condition during heating with the burner—was it reductive or oxidative?

 Generally, there is a possibility of excess aluminum entering the silicon, which has not been considered. Is the produced metal always pure without aluminum in it?

 I am not an expert in CFD calculations, but verification is usually required from simulation calculations using CFD (Computational Fluid Dynamics), for example, using a water model!

Is the study design appropriate and does the work have academic merit?

Partly

Is the work clearly and accurately presented and does it cite the current literature?

Partly

If applicable, is the statistical analysis and its interpretation appropriate?

Not applicable

Are all the source data underlying the results available to ensure full reproducibility?

Partly

Are the conclusions drawn adequately supported by the results?

Partly

Are sufficient details of methods and analysis provided to allow replication by others?

Partly

Reviewer Expertise:

High temperatures metals processing, ironmaking & steelmaking, ferronickel smelting, tin smelting, magnesium production by metallothermic, carbothermic reduction, hydrogen and methane reduction, hydrogen plasma smelting reduction.

I confirm that I have read this submission and believe that I have an appropriate level of expertise to confirm that it is of an acceptable scientific standard, however I have significant reservations, as outlined above.

# Response

**Published:** 2024-12-21

**Authors:** Iván Martínez Suárez

**Affiliations:** Matemática Aplicada, Universidade de Santiago de Compostela, Santiago de Compostela, Galicia, Spain

We want to thank the reviewer for reading the manuscript so carefully and for the given remarks, which we believe allowed us to improve the paper. In what follows we briefly comment the suggestions and questions that were made.

- *The manuscript does not provide information regarding the temperature of the silicon separation process and the CaO-Al₂O₃ slag, including viscosity and density data of silicon and CaO-Al₂O₃ slag at what temperature? What are the data sources for Table 1 and Table 2?*

The temperature considered for the mixture of slag and silicon is about 1400 ºC. Viscosity and density data at that temperature are shown in Table 1 and were provided by our experimental partners in the SisAl Project. This point has been clarified in the text. Concerning Table 2, different scenarios are proposed for simulation purposes. The material properties in Table 2 which are not included in Table 1 are only for simulation and comparison between scenarios.  

- *What was the environmental condition during heating with the burner—was it reductive or oxidative?*

The furnace at FRey facilities is equipped with an oxy-combustion natural gas burner using pure oxygen as oxidizer.  

- *Generally, there is a possibility of excess aluminum entering the silicon, which has not been considered. Is the produced metal always pure without aluminum in it?*

In different experimental tests in plant, some impurities were observed in the final produced silicon, which would require further refining processes to achieve optimal purity. Of course, these impurities could affect the final separation time of the components of the mixture, although our aim in this work is to make an initial approximation from a less detailed perspective. This point was clarified in the introduction.  

- *Verification of simulation calculations using CFD (Computational Fluid Dynamics), for example, using a water model.*

It would be optimal to be able to measure the separation time in plant, but this is difficult to do accurately due to the daily production needs of our industrial partner and further work would be needed. Nevertheless, the results obtained with the Eulerian model are in line with the results of close observation in plant.

# Reviewer report: Reviewer response for version 1

**DOI:** 10.21956/openreseurope.20381.r46993

**Published:** 2024-12-16

**Authors:** Navneet Singh Randhawa

**Affiliations:** 1 CSIR-National Metallurgical Laboratory, Jharkhand, India

The manuscript reports the numerical simulation work on the Separation Time of Aluminothermic Reduction Products for Sustainable Silicon Production. This work is industrially relevant and would interest researchers in this field. The manuscript may be indexed after revision with the following comments.

 The relevant literature on silicon reproduction should be thoroughly presented and compared with the present work.

 The economic aspect of the aluminothermic process is lacking in the manuscript. A bride account may be given.

 The method may be elaborated so that the researched would understand and follow the approach.

 The optimal separation time determined by the model should be mentioned and discussed.

Is the study design appropriate and does the work have academic merit?

Yes

Is the work clearly and accurately presented and does it cite the current literature?

Partly

If applicable, is the statistical analysis and its interpretation appropriate?

I cannot comment. A qualified statistician is required.

Are all the source data underlying the results available to ensure full reproducibility?

No

Are the conclusions drawn adequately supported by the results?

Yes

Are sufficient details of methods and analysis provided to allow replication by others?

Partly

Reviewer Expertise:

Valorization of metallurgical wastes and by-products. Computational thermodynamics for Extractive Metallurgy.    Extraction of metal values from primary and secondary. resources. Pilotscale evaluation of metal extraction processes

I confirm that I have read this submission and believe that I have an appropriate level of expertise to confirm that it is of an acceptable scientific standard, however I have significant reservations, as outlined above.

# Response

**Published:** 2024-12-21

**Authors:** Iván Martínez Suárez

**Affiliations:** Matemática Aplicada, Universidade de Santiago de Compostela, Santiago de Compostela, Galicia, Spain

We want to thank the reviewer for reading the manuscript so carefully and for the given remarks, which we believe allowed us to improve the paper. In what follows we briefly comment the suggestions that were made.

- *The relevant literature on silicon reproduction should be thoroughly presented and compared with the present work.*

Information regarding this issue has been enriched and completed in the article introduction, including the addition of relevant references on silicon production.  

- *The economic aspect of the aluminothermic process is lacking in the manuscript. A bride account may be given.*

The economic aspects of the aluminothermic process have mainly been addressed by specialized partners of the SisAl Pilot Project. Nevertheless, in pooling the conclusions of the different partners, it was agreed that the production of silicon through aluminothermic reduction would be feasible on an industrial scale both from a technical and an economic point of view. A remark was added in the introduction to clarify this point.  

- *The method may be elaborated so that the researched would understand and follow the approach.*

Our aim when describing the methods was to provide a guide that regular users who are familiar with CFD simulation could follow, including standard mathematical models in a 2D computational domain. The simulations were performed by using the software Ansys Fluent 2022 R2 and the accessible open source alternative, OpenFOAM, was also recommended.  

- *The optimal separation time determined by the model should be mentioned and discussed.*

The separation time of the slag and the silicon is analysed under different simulation scenarios, including different material properties, initial conditions and multiphase models. As an insight of these simulations, the Eulerian model is the one that best fits the separation times observed in plant. For industrial purposes it would the best to have a separation time as short as possible, but our aim in this work is only to study the separation time due to density differences and viscosity effects. A further step could involve the analysis of novel separation methods that could eventually optimize the separation time. A remark has been added to the conclusions to clarify this point.
